# Uncovering drivers of climate research in policy with pretrained language models

**DOI:** 10.1016/j.patter.2025.101342

**Published:** 2025-08-07

**Authors:** Basil Mahfouz, Licia Capra, Geoff Mulgan

**Affiliations:** 1 UCL Department of Science, Technology, Engineering and Public Policy, London, UK; 2 UCL Computer Science, London, UK

**Keywords:** climate policy, research evaluation, natural language processing, machine learning, near-miss analysis, citation patterns, bibliometric indicators, evidence-based policy, knowledge transfer, science-policy interface

## Abstract

Evidence-based policymaking is crucial for addressing societal challenges, yet factors driving research uptake in policy remain unclear. Previous studies have not accounted for the confounding effect of policy relevance, potentially skewing conclusions about impact drivers. Using climate change as a case study, we employ pretrained language models to identify semantically similar research paper pairs where one is cited in policy and the other is not, controlling for inherent policy relevance. This approach allows us to isolate the effects of various factors on policy citation likelihood. We find that in climate change, academic citations are the strongest predictor of policy impact, followed by media mentions. This computational method can be extended to other variables as well as different scientific domains to enable comparative analysis of policy uptake mechanisms across fields.

## Highlights

• Computational method for analyzing research policy uptake drivers at scale
• Semantic matching controls for content effects in science-policy studies
• Academic citations strong predictors of climate research policy uptake

## The bigger picture

Climate change, pandemics, and countless other challenges require societies to effectively integrate knowledge into decision-making, yet why some valuable research reaches policymakers while other important work remains unused is largely unknown. Using natural language processing, we identify thousands of semantically similar research papers where one is cited in policy and the other is not, creating the experimental-like conditions needed to isolate what really drives knowledge uptake.

Our climate research case study demonstrates the approach and reveals that academic citations and media attention drive policy uptake more than government collaboration or author geography. Researchers can use the presented pairing method to investigate any factors they believe might influence policy impact, from author networks to research methodologies, while controlling for the confounding effects of content relevance. As societies become ever more dependent on research to navigate complexity, such methods offer opportunities for diagnosing and improving the systematic processes that determine how our collective knowledge influences critical decisions.

## Teaser

This study examines why some climate research influences policy while similar research does not. Using natural language processing to match semantically similar papers, the authors found that academic citations and media mentions strongly predict policy uptake, even among equally relevant research. These findings reveal systematic selection processes that may delay important climate science from reaching policymakers. The method can be applied to study other factors influencing research-policy connections.

## Introduction

Effective policymaking for complex challenges depends critically on scientific knowledge, yet we lack a clear understanding of why some research influences policy while other work is overlooked. While the science-policy interface has received considerable scholarly attention, particularly in climate change,^1^^,^^2^^,^^3^^,^^4^^,^^5^ quantitative analysis at scale has only recently become possible with comprehensive policy databases like Overton^6^ and bibliometric metadata. However, emerging quantitative studies face methodological challenges due to confounding effects of policy relevance, or the inherent tendency for certain research topics to be more applicable to policy challenges.

Drawing on Weiss’s^7^ models, research varies in the extent to which it provides conceptual insights, reframes policy challenges, or offers actionable knowledge that can inform policy thinking. This latent characteristic of policy relevance, reflecting the inherent salience of research topics to policymakers, inherently affects which papers get cited in policy. Research addressing topics that are applicable to policy challenges, whether in the form of empirical studies, methodological papers, or literature reviews, is more likely to be cited regardless of other factors.

Yet, despite this variability in research relevance, early quantitative studies exploring how different indicators affect the likelihood of research being cited in policy documents^8^^,^^9^^,^^10^^,^^11^^,^^12^^,^^13^ treated non-cited research as a homogeneous category, not differentiating between papers genuinely irrelevant to policy and those overlooked despite high relevance, raising questions about possible omitted variable bias.

There is already some evidence to the possible effect of policy relevance. For example, Bornmann et al.^8^^,^^9^ found only a modest correlation between policy citations and scientific citations in climate change research, indicating that policy and academic circles often prioritize different sets of papers. Mahfouz et al.^14^ identified that research aligned with the United Nations sustainable development goals (SDGs), which tends to be more relevant to policymakers, is cited in policy documents nearly five times more than non-SDG-related research. Similarly, Pinheiro et al.^15^ demonstrated that cross-disciplinary research, which is often more relevant to policy, is more frequently cited in policy documents. Perhaps most compelling, Nelson et al.^16^ showed that deep learning models could predict the inclusion of research in policy documents based on content more accurately than other bibliometric features.

The issue of confounding effects due to research content has been highlighted in the literature. To control for content-based effects, Noyons^17^ proposed to shift analysis from individual publications to research areas defined by their societal connectedness, with significant potential for studying the science-policy interface.^18^ Similarly, in the research commercialization literature. For instance, factors such as “latent patentability” and “latent commercializability” of science have been shown to significantly affect the likelihood of scientific discoveries being brought to market.^19^^,^^20^^,^^21^^,^^22^ Marx and Hsu^22^ addressed this by controlling for commercial potential through the identification of “research twins,” or pairs of papers with equal commercial potential, allowing them to isolate the effects of their studied indicators from the confounding influence of research content.

In this study, we develop a scalable computational method to understand what drives the incorporation of scientific research into policy across research domains, using climate change as a case study. We adapt Marx and Hsu’s^22^ approach of finding research paper pairs, using a pretrained language model, to identify “near misses,” or pairs of highly similar climate research papers with equivalent policy relevance but different policy citation outcomes. Using semantic similarity embeddings from the MiniLM language model, we matched 10,293 pairs of highly similar climate research papers, where one was cited in policy documents and the other was not. By analyzing these matched pairs using conditional logistic regression, we examined how academic citations, journal CiteScore, author h-index, government collaboration, authors’ past policy citations, media mentions, and geographic origin influence policy citation.

This study contributes to the literature in several ways. First, it provides a deeper understanding of the drivers of scientific uptake in climate policy by controlling for content-based confounding effects, helping identify systemic bottlenecks and providing guidance on which characteristics of papers influence policy citation. Using this dataset and approach, other research characteristics related to methodology, scope, and content can be explored, such as whether papers contain models and data analysis or are primarily conceptual and whether the research focuses on single or multiple countries. Such extensions could leverage large language models to extract methodological features from papers, enabling systematic analysis of how different types of empirical approaches influence policy uptake.

Second, it complements an array of natural language processing (NLP)-based research exploring the science-policy interface. These include tools mapping research priorities to policy needs,^23^ assessing evidence quality in policymaking,^24^ mapping climate policy priorities and gaps,^25^^,^^26^^,^^27^^,^^28^^,^^29^^,^^30^ and exploring policy interventions at scale.^31^ Our approach adds to this suite by examining the upstream selection mechanisms that determine which science gets cited in policy. While existing quantitative studies^8^^,^^9^^,^^10^^,^^11^^,^^12^ have explored what gets cited in policy, our method provides a scalable approach for understanding citation drivers while controlling for policy relevance, addressing the omitted variable bias that has limited previous research on science-policy uptake.

### Choice of metrics

The first theme examines traditional bibliometric indicators that track academic influence and visibility. Previous studies^8^^,^^9^^,^^10^^,^^11^^,^^18^ have reported varying connections between these indicators and research policy impact. Our study reassesses these relationships while controlling for policy relevance, seeking to understand if academic citations and impact serve as a pathway to policy citation.

The first indicator is academic citation count, which measures how frequently a paper has been referenced in other scholarly works. For each pair, we included citations accrued up to the date of the citing policy document’s publication. As our dataset focuses on climate change papers matched by content and publication date, no field or year normalization was needed.

The second indicator is the publishing journal’s CiteScore, calculated by dividing citations a journal receives in a year by the number of documents it published in the preceding three years. This metric reflects a journal’s visibility within academic discourse. For each paper pair, we recorded the CiteScore at the year of policy citation.

The third indicator is author h-index, a composite measure of both productivity and citation impact of a researcher’s body of work. For each paper, we calculated co-authors’ h-indexes at the time of policy citation, selecting the highest value among the authoring team. While average h-index across all authors might seem more intuitive, we chose maximum h-index based on the rationale that policymakers are more likely to be influenced by the presence of at least one highly established researcher on a team rather than the average standing of all authors, particularly given that average h-index can be substantially reduced by early-career researchers or graduate students. To ensure robustness, we also tested our analysis using average h-index.

The second theme examines cross-sector collaboration between researchers and government. Building on co-production literature, which highlights how stakeholder involvement enhances research relevance and impact,^32^^,^^33^^,^^34^ we measured formal institutional connections through co-authorship as an indicator of cross-sector partnership. Using SciVal^35^ affiliation data, we assigned each paper a binary score indicating whether at least one co-author is affiliated with a government organization. This allows us to test whether papers with direct academic-government collaboration are more likely to be cited in policy documents.

The third theme examines the policy experience of authors through their citation history. For each author, we counted previous papers cited in policy documents that were published before the policy document in our near-miss dataset. This metric assesses whether researchers with established policy impact are more likely to have their new work cited in policy. Oliver et al.^36^^,^^37^ found that policymakers tend to return to trusted sources, suggesting authors with prior policy citations may develop effective communication skills, build institutional relationships, and establish reputations as reliable evidence sources.

The fourth theme of metrics is based on the literature that argues that traditional academic publications are not an effective medium for reaching policymakers.^38^^,^^39^^,^^40^^,^^41^ We tested whether alternative dissemination methods, such as mentions in news and social media, increased the likelihood of a paper being cited by policymakers. To do this, we focused on altmetric scores, which track how often a paper is referenced on platforms like blog posts, news articles, and Wikipedia, as curated by PlumX.^42^

Finally, the fifth theme explores the impact of researcher geography. Given the systemic inequalities and underrepresentation faced by Global South researchers, particularly in climate change and environmental sciences,^43^^,^^44^^,^^45^ we investigated whether a researcher’s geographic affiliation influences the likelihood of their work being cited in climate policy. We assigned a binary score to each paper, indicating whether at least one co-author is from a Global South country as designated by the United Nations Trade and Development.

## Results

**Figure 1:**
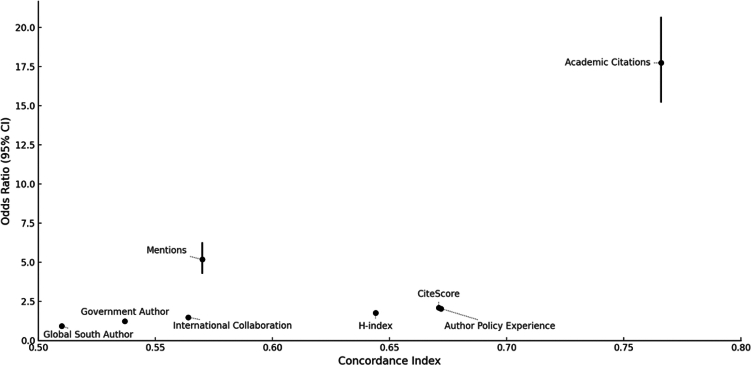
Comparison of model performance (concordance) and effect sizes (coefficients) for different predictors of policy citation Error bars on coefficients show 95% confidence intervals.

All examined indicators significantly influenced the likelihood of a paper being cited in policy. Except for the Global South authorship indicator, all metrics demonstrated a positive relationship with policy impact. Figure 1 visualizes the results from each individual model.

### Academic citation counts

**Figure 2:**
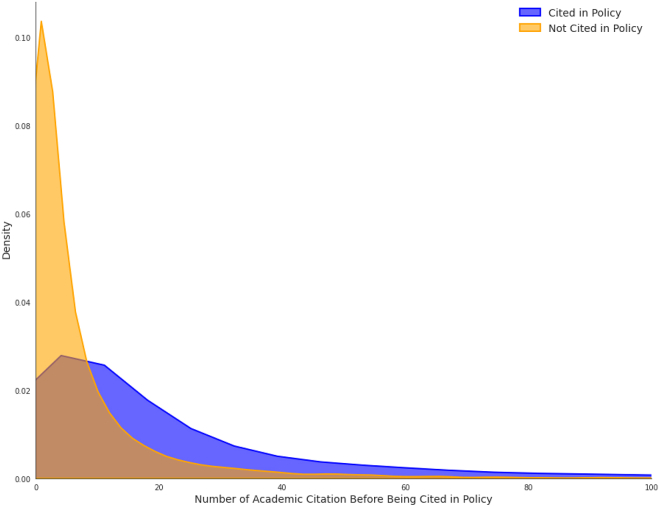
Distribution of academic citations

Our analysis reveals that academic citations accrued before policy publication are the strongest predictors of policy citation among similar, policy-relevant papers. The distribution of academic citation counts, as shown in Figure 2, reveals a large difference between papers cited in policy and those not cited. On average, at the date of policy citation, the papers cited in policy received 3.6 times more academic citations (*n* = 24) compared to their near-miss counterparts (*n* = 6.6). Among the 10,293 pairs analyzed, 72.8% of the cited papers had higher academic citations, 7.6% had equal citations, and in the remaining 19.6% of cases, the non-cited paper had higher academic citations.

The conditional logistic regression model shows a strong relationship between adjusted citation count and policy citation likelihood. Each one standard deviation increase in adjusted citation count raised the odds of policy citation by approximately 17.75 times (95% CI [15.22, 20.69], *p* < 2e−16). The model demonstrates good predictive capability with a concordance index of 0.766 (SE = 0.006).

### Journal CiteScore

For CiteScore, which calculates the mean citations per article of a journal within a defined time window, as a result of sparse data, not all papers could be matched to journals with a calculated CiteScore for the year of policy publication. As a result, pairs were excluded if either paper lacked a CiteScore, leaving 7,632 near-miss pairs for analysis.

**Figure 3:**
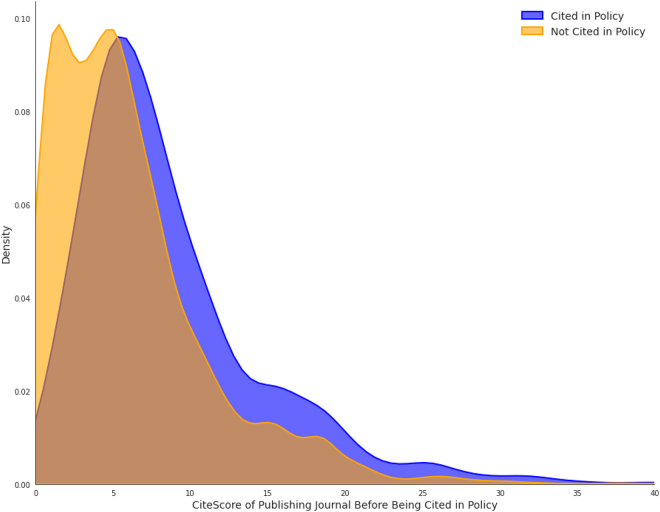
Distribution of CiteScore

On average, policy-cited papers had a CiteScore 1.49 times higher (*n* = 9.14) than their non-cited counterparts (*n* = 6.15). Figure 3 shows the distribution of CiteScores between the policy-cited papers and their near-miss counterparts. Among the pairs, 63.6% of policy-cited papers had a higher CiteScore, 7% had equal scores, while in 29.4% of pairs, the non-cited paper had a higher CiteScore.

The conditional logistic regression model reveals a statistically significant relationship between CiteScore and policy citation likelihood. Each one standard deviation increase in CiteScore raises the odds of policy citation by approximately 2.1 times (95% CI [1.996, 2.224], *p* < 2e−16). The model demonstrates moderate predictive capability with a concordance index of 0.671 (SE = 0.007).

### Author h-index

**Figure 4:**
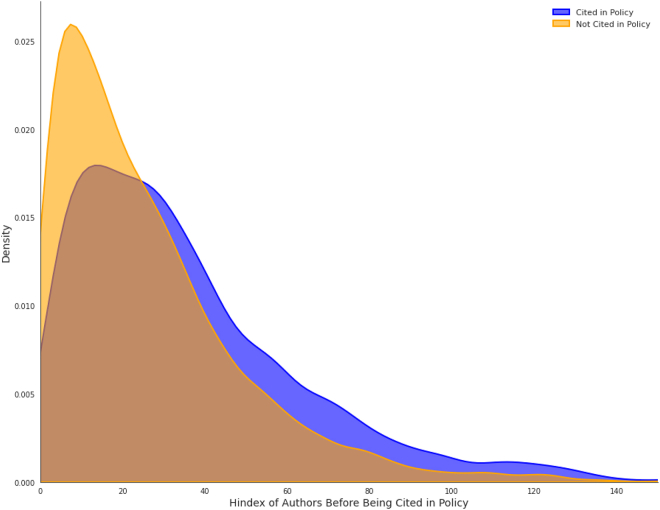
Distribution of h-index

The analysis of 10,277 pairs of author h-indexes shows that papers cited in policy had a 40.75% higher maximum h-index (*n* = 37.2) compared to those not cited (*n* = 26.5). The distribution of maximum h-index values is depicted in Figure 4. In 63.4% of the pairs, the cited paper had a higher h-index, while 2% of pairs had equal h-index values. In the remaining 34.6% of pairs, the non-cited paper had a higher h-index.

The conditional logistic regression model reveals a significant association between h-index and policy citation likelihood. Each standard deviation increase in h-index raises the odds of policy citation by approximately 1.76 times (95% CI [1.695, 1.837], *p* < 2e−16). The model demonstrates moderate predictive ability with a concordance index of 0.644 (SE = 0.007). Analysis using average h-index confirmed this relationship with similar effect direction and statistical significance (odds ratio = 1.64, 95% CI [1.575, 1.699], concordance = 0.627).

### Collaboration with government affiliated authors

Analysis of 8,763 near-miss pairs with institutional data revealed that 74% of papers were authored by academic-only teams. Only 32% of pairs showed a difference in government co-authorship status (one paper having government collaboration while the matched paper did not), limiting our ability to isolate this effect within the matched-pair design. Within these mixed pairs, in 61% of cases, the government-authored paper was the one cited in policy.

The conditional logistic regression model reveals a significant positive association between government collaboration and policy citation likelihood. Papers with at least one government-affiliated author have approximately 22.8% higher odds of policy citation (exp(coef) = 1.228, 95% CI [1.188, 1.269], *p* < 2e−16). While statistically significant, the model shows weak predictive ability with a concordance index of 0.537 (SE = 0.004).

### Author past policy impact

**Figure 5:**
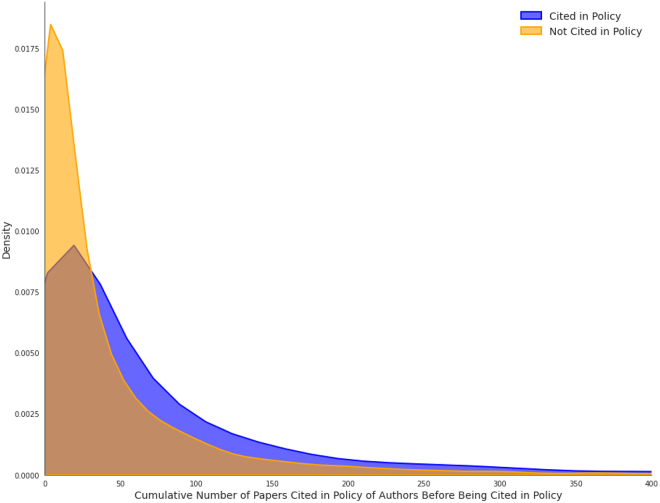
Distribution of cumulative policy citations

On average, authors of the policy-cited papers had double the number of past papers cited in policy (*n* = 75.4) than their near-miss counterparts (*n* = 38.6). Within pairs, there is also a strong variation. In 65.6% of pairs, the authors of papers cited in policy had more prior papers cited in policy than the authors of their near-miss counterpart, 3.24% had equal past policy experience, and in 31% of pairs, the near-miss authors had more prior policy citations. It is important to note that while we filtered the authors’ papers to include only those published before the policy document that cites the paper in the near-miss/cited pair, we cannot identify whether these earlier papers were cited by policy before or after the policy document in question. However, since the papers were published before the cited paper, we assume that they were more likely to have been cited before the policy document was published. The distribution is visualized in Figure 5.

The conditional logistic regression model demonstrates a significant association between authors’ prior policy citations and policy citation likelihood. Each standard deviation increase in authors’ past policy experience doubles the odds of policy citation (exp(coef) = 2.039, 95% CI [1.921, 2.163], *p* < 2e−16). The model shows moderate predictive ability with a concordance index of 0.672 (SE = 0.006).

### Media mentions

**Figure 6:**
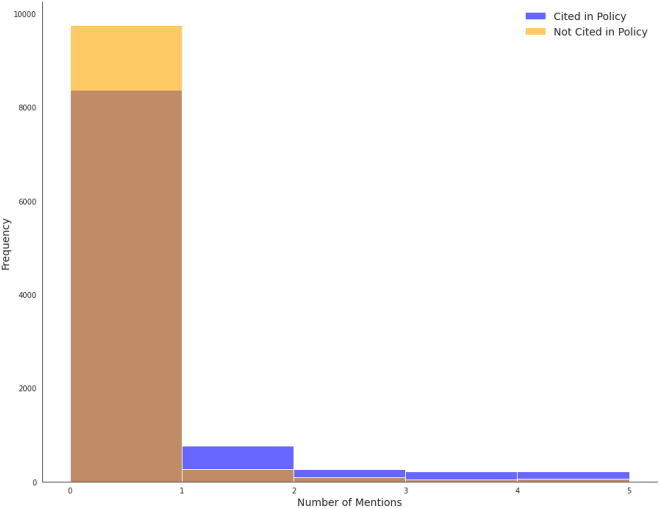
Mentions histogram

Mentions in the media were rare across both cited and non-cited sets, with 78.31% of pairs having equal mentions, most of which were zero. However, cited papers had an average of 1.29 mentions, 7.59 times higher than for non-cited papers. In 17.8% of pairs, the cited paper had a higher mention count, while in 3.89% of pairs, the non-cited paper had more mentions. Figure 6 shows the distribution of media mentions across cited and non-cited papers. It is important to note that mentions are cumulative and include those made after the citing policy publication date. ICSR Lab only provides counts of mentions without the specific dates or metadata on the mentioning media.

The conditional logistic regression model shows a strong association between news mentions and policy citation likelihood. Each standard deviation increase in news mentions raises the odds of policy citation by approximately 5.18 times (95% CI [4.271, 6.276], *p* < 2e−16). The model demonstrates moderate predictive ability with a concordance index of 0.57 (SE = 0.003).

### Global South authorship

When studying the influence of geography, sparce data prevented tracking some authors’ affiliations or country. Pairs were dropped if one paper lacked geography information for all authors. This left 8,625 final pairs for geographical analysis, with 77% of papers authored exclusively by Global North institutions.

The conditional logistic regression model shows that having at least one co-author from the Global South reduces the odds of policy citation by approximately 7.4% (exp(coef) = 0.926, 95% CI [0.892, 0.963], *p* = 9.23e−05). Although statistically significant, the model demonstrates very weak predictive ability with a concordance index of 0.51 (SE = 0.004).

### International collaboration

We analyzed the number of countries represented in each paper’s authorship. Analysis of the 8,625 pairs showed equal country representation in 52.6% of pairs. In 30.1% of pairs, the cited paper had authors from more countries, while in 17.3% of pairs, the non-cited paper had greater international representation.

The conditional logistic regression model demonstrates a statistically significant relationship between international collaboration and policy citation likelihood. Each one standard deviation increase in the number of countries raises the odds of policy citation by approximately 48.8% (exp(coef) = 1.488, 95% CI [1.423, 1.555], *p* < 2e−16). To put this in practical terms, each additional country in the authorship team increases the odds of policy citation by approximately 38.6%. The model shows moderate predictive ability with a concordance index of 0.564 (SE = 0.005). This finding suggests that international collaboration enhances policy visibility, as multi-country research addresses climate challenges with broader geographic applicability than semantically similar single-country studies.

### Multicollinearity check

**Figure 7:**
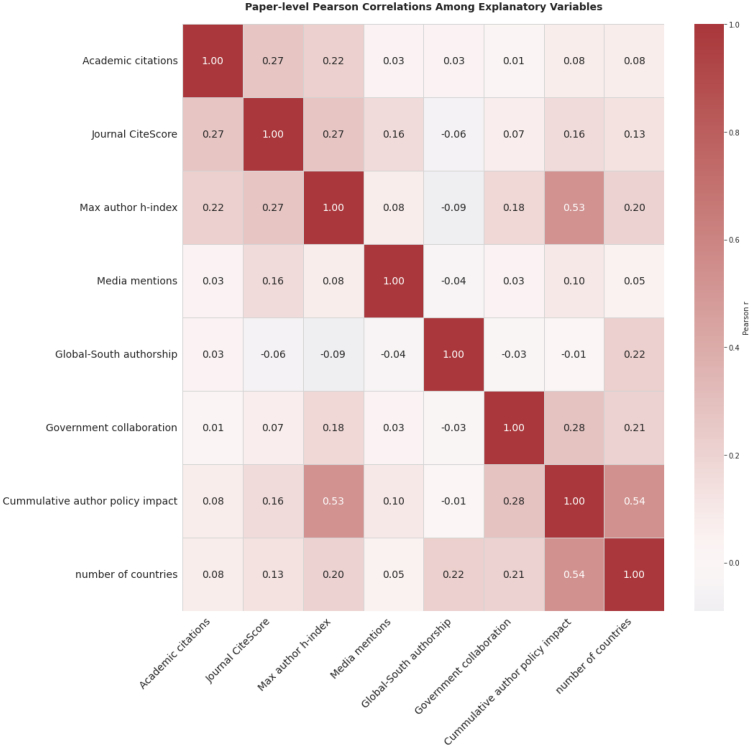
Correlation matrix among all variables

We examined correlations between predictors to assess whether variables operate independently or through shared mechanisms. The correlation matrix in Figure 7 reveals generally weak correlations among explanatory variables, with most coefficients below 0.30. This suggests each predictor contributes relatively independent explanatory power rather than measuring overlapping constructs.

The strongest correlations occur between author h-index and cumulative policy impact (r = 0.64) and between number of countries and cumulative policy impact (R= 0.55). These reflect expected relationships where established researchers with prior policy influence maintain international collaborations and continued policy engagement. Media mentions demonstrate consistently weak correlations (R= 0.03–0.16), indicating independent operation from other metrics.

To confirm these correlations do not compromise regression estimates, we calculated variance inflation factors (VIFs). All values ranged from 1.00 to 1.85, well below the threshold of 5.0, indicating no problematic multicollinearity.^46^ This validates our conditional logistic regression approach and confirms each predictor’s estimated effects remain statistically reliable despite the moderate correlations observed.

## Discussion

This study reveals important insights into the mechanisms driving scientific research uptake in climate policy documents. By using pretrained language models to identify semantically similar papers where one is cited in policy and one is not, we were able to control for the inherent policy relevance of research content. Our findings demonstrate that even among equally policy-relevant research papers, significant differences in policy citation likelihood exist, driven primarily by non-content factors.

Policymakers predominantly rely on paper-level indicators rather than journal or author-level metrics. This may stem from two mechanisms: policy staff with academic backgrounds actively seeking validated research or using citations as a signal of scholarly credibility. In the context of climate change research, with its robust science policy interface through the Intergovernmental Panel on Climate Change (IPCC), citations likely represent a more rigorous evaluation process.

However, this citation-driven approach presents significant methodological challenges. Citation metrics vary substantially across disciplines, potentially creating systemic biases that marginalize innovative or locally relevant research. Citations accumulate slowly, which can delay integrating urgent research into policy decisions. Moreover, early citation counts may not reliably predict long-term impact, as papers can gain rapid initial attention but subsequently lose influence.

Our analysis exposes citation metrics as heuristics that actively filter scientific perspectives, potentially constraining the diversity of evidence in climate policy debates. For researchers, this can incentivize conservative publication strategies; for policymakers, uncritical reliance on citations risks overlooking crucial scientific contributions.

Our analysis also confirmed that both maximum and average h-index approaches yield consistent conclusions about the positive relationship between author reputation and policy citation likelihood, with maximum h-index providing marginally stronger predictive power (concordance 0.644 vs. 0.627). This suggests that the reputation of the leading author matters more than overall team standing for policy citation outcomes.

Mentions in the media rank as the second most important factor (in terms of odds ratio) in determining policy impact. Although over 78% of the near-miss pairs in the study had the same number of mentions, usually zero, when media mentions do occur, they greatly increase the likelihood of research being cited in policy documents. This suggests that while media mentions are not necessary for policy impact, they can significantly amplify the reach and influence of research beyond academia. However, this raises a concern that researchers or institutions with stronger media connections may have an undue advantage in shaping climate policy. It could potentially skew the policymaking process toward research that gains media attention over more robust research that remains less visible.

Our analysis shows that papers with government affiliated co-authors were only marginally more likely to be cited in policy documents when controlling for policy relevance. Although co-authorship serves as a measurable indicator of researcher-policymaker engagement, it represents just one visible form of interaction between science and policy. Our measure cannot capture other important engagement types such as stakeholder workshops, advisory roles, or informal knowledge exchange. Furthermore, while the weak relationship between co-authorship and policy impact appears to challenge conventional literature suggesting that co-production or engaging with policymakers is critical for policy impact, the weak relationship may also reflect our methodology, which examines papers that are already highly policy relevant in content, where the additional visibility gained through government co-authorship provides less advantage than might be observed. Government collaboration may primarily enhance policy relevance during development, but once relevance is achieved, formal co-authorship itself provides minimal influence on the likelihood of policy citation.

Additionally, our binary government collaboration measure treats all government-affiliated co-authors as equivalent despite differences in their proximity to policymaking. This institutional heterogeneity may attenuate the observed relationship between co-authorship and policy citation. Future research employing granular institutional classification or examining specific author roles within government could reveal stronger associations between collaboration type and policy uptake.

Similarly, papers authored by researcher teams with a greater number of prior policy citations were more likely to be cited in policy. Given the highly skewed nature of policy citations in climate research, where 10% of authors contribute 96% of cited papers, this result was unexpected. One explanation is the composition of our dataset, which includes authors with significantly more prior policy citations than the average climate change researcher. Specifically, the average climate change paper has 14.35 policy-cited papers per author team, representing only 18.5% of the cumulative prior papers cited in policy for the authors of the cited papers in our dataset and 37% for the near-miss uncited papers. This suggests that both cited and near-miss papers were produced by researchers already more engaged with policy, making the additional effect of prior policy impact marginal. It also suggests that once a certain level of policy impact is reached, additional impact may not be essential for increasing policy citation likelihood.

**Figure 8:**
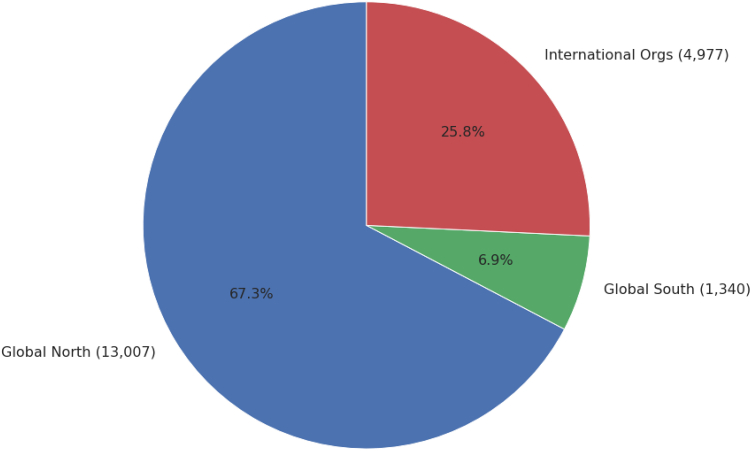
Number of citing policy documents per region

Finally, we found that the inclusion of authors from Global South institutions slightly decreased the likelihood of their papers being cited in policy documents. However, the low concordance index (0.51) for this metric suggests that geographic origin alone is a very weak policy impact, slightly better than random chance. This observed trend may be influenced by the coverage limitations of the Overton policy database, shown in Figure 8, which predominantly includes policy documents from Global North countries.^9^^,^^47^ In our near-miss dataset, only 7% of the citing policy documents originated from Global South countries. Combined with evidence that policymakers often prefer to cite local researchers,^48^^,^^49^ this imbalance likely contributes to the observed negative effect on citations for Global South authors.

### Limitations of the study

With over 20,000 research papers analyzed, this study represents one of the first, largest, and most comprehensive empirical studies of the factors influencing the uptake of climate change research in policy. We employ cutting-edge computational methods, including large language models, and rely on new, rapidly evolving datasets. However, there are limitations inherent in this type of large-scale analysis, with the structure, methods, and assumptions playing a key role in framing how we understand the results.

First, the results must be understood within the strict experimental context of our methodology. By focusing on pairs of similar, and highly policy-relevant papers, we controlled for the confounding effect of policy relevance. However, this matching approach inevitably excludes papers that lack suitable matches, potentially overlooking highly innovative or groundbreaking research and important dynamics at the frontier of research. These unique contributions may follow different pathways to policy impact that our analysis cannot capture.

Similarly, our analysis necessarily excludes recent publications (after 2022) due to insufficient time for policy citation accumulation. This temporal constraint means we cannot capture how cutting-edge climate research enters policy discourse before traditional academic validation through citations. Future research could examine recently cited papers by pairing them with similar, recently published but uncited work, though such analysis faces the challenge of finding sufficient semantically similar papers for newer research. This approach would complement our findings by revealing whether different mechanisms drive policy uptake for emerging versus established research.

Second, while our methodology creates cognate pairs with strong semantic overlap, these represent plausible rather than perfect experimental controls. Perfect counterfactuals are methodologically impossible in observational research. Identical papers would constitute duplicate publication, which is editorial malpractice. Our approach offers a scalable alternative to Marx and Hsu’s^22^ manually identified research twins that is more robust at studying bibliometric drivers of policy citation than conventional field-wide bibliometric studies that lack content controls.^8^^,^^9^^,^^10^^,^^11^^,^^12^

Third, while our methodology leverages pretrained language models to identify semantically similar papers, these embedding models have their own limitations when applied to specialized scientific domains. Though MiniLM demonstrated strong performance in our comparative testing against other models, it was primarily trained on general text rather than specifically on climate science terminology. This may introduce subtle biases or inconsistencies in semantic matching, particularly for highly technical abstracts with domain-specific vocabulary or for emerging research areas with evolving terminology. While manual validation of 50 randomly selected pairs confirmed semantic similarity with zero false positives, this validation was conducted by a single researcher rather than multiple independent reviewers. Future applications would benefit from domain-specific models and multi-reviewer validation with inter-rater reliability metrics to strengthen confidence in matching quality and scalability across subdisciplines.

Fourth, while Scopus requires English abstracts and titles for all indexed content, we cannot rule out that some authors may have used machine translation tools when preparing these English versions from their original language manuscripts. If such automated translations were used, they could introduce subtle inconsistencies or inaccuracies in technical climate science terminology that might affect the quality of our semantic similarity matching. However, only 411 unique papers of the total 18,297 unique papers in our final matched set were originally published in another language, meaning this potential limitation affects at most 2% of our dataset. This represents a limitation inherent to any large-scale study using Scopus metadata rather than a methodological choice in our research design.

Fifth, while Overton, the database we used to track policy citations, is one of the largest policy databases available, it does not capture all policy documents. Although policy representation from the Global South is growing, the database remains dominated by policy documents from primarily English-speaking, Global North countries.^9^^,^^47^ Additionally, Murat et al.^50^ found that only 23.1% of organizations tracked by Altmetric.com, another policy database, are also indexed in Overton, with only 7% of Overton-listed organizations appear on Altmetric.com. Future research could replicate and refine our findings by using both databases.

Sixth, we relied on Elsevier’s SDG classifications to identify climate change research.^51^ While this method is effective, different approaches to mapping research to the UN SDGs exist, each yielding varying results.^52^^,^^53^ Climate change is also highly interdisciplinary, and a significant portion of climate research may be classified under other SDGs, such as SDG 7, “affordable and clean energy”; SDG 11, “sustainable cities and communities”; SDG 14, “life below water”; and SDG 15, “life on land.” Different scoping methodologies could result in different datasets, potentially affecting the outcomes of studies like ours. By focusing our queries on SDG 13 papers, we concentrated on the literature most directly related to climate change, the core research in this field. To mitigate potential bias from SDG classification issues, we conducted the matching k-nearest neighbors (KNN) search on the entire Scopus dataset. While our queried papers were originally from the core set of SDG 13 climate change research, they were semantically matched to papers across the entire Scopus database, capturing a broader range of related research.

Seventh, within our dataset, 18% of papers formed larger clusters through interconnected matches. We chose to analyze each pair independently of its cluster, which may have introduced some bias. However, we estimate this bias to be minimal, as most of these clusters had only one additional paper, with only 2% of our dataset belonging to clusters of more than three papers. Similarly, while most cited papers had only one near-miss match, around 17% of cited papers had more than one match, with 15 outliers having more than five matches. In this analysis, each cited/near-miss match was treated as an independent pair, even when a cited paper had multiple matches. This approach allowed for a simpler, cleaner analysis despite the duplication, though future studies could address this by incorporating methods that account for the network effect.

Eighth, some authors lacked institutional affiliation data, which affected the analysis on geography and government affiliation. During the data-filtering process, we excluded pairs of papers where all authors of either paper were missing affiliation information. However, if at least one author from a paper had affiliation data, we retained that paper for analysis. While this approach may have introduced bias, it was counteracted by allowing us to retain as much data as possible.

Finally, while we were able to reduce noise by calculating CiteScore, academic citations, and h-index at the time of policy publication, we were not able to do so for mentions and team prior policy citations. Specifically, we could not limit media mentions and prior policy citations to those accrued before the policy citation date, making it unclear whether they occurred before or after the paper was cited in policy. For authors’ prior policy experience, we minimized this effect by including only papers published before the policy date of the cited paper. However, we could not determine if these earlier papers were cited in policy documents before or after the policy citation in question. This limitation is a common issue in bibliometric research, which typically consider total citation counts without accounting for when citations occur relative to certain events. Despite this, our approach of calculating certain metrics at the time of policy publication helps reduce some of the noise, providing a more accurate snapshot of the information available to policymakers at that time.

## Methods

Bibliometric data for this study was sourced from Elsevier’s International Centre for the Study of Research^54^ and policy citation data via Overton.^6^ ICSR provides metadata on over 90 million Scopus-indexed scholarly papers up to March 29, 2024, while Overton indexes policy documents and their citations to scholarly research. By using both databases, we can trace the influence of research on policymaking and study the characteristics of the citing policy documents and cited scholarly research.

Scholarly papers in the ICSR Lab database are categorized into one of the 17 United Nations SDGs based on the methodology of Bedard-Vallee et al.^51^ Given the focus of our study on climate change research, we identified 201,270 scholarly articles related to SDG 13, “climate action,” published between 2016 and 2021, of which around 18% are cited in policy documents indexed in Overton. We excluded articles published after 2021, as they may not have had enough time to gather policy citations, consistent with trends in policy citation timelines identified by Szomszor and Adie.^47^

### Model selection

To identify highly similar articles, a KNN search was performed using Elasticsearch^55^ on abstract embeddings from 20 million articles, generated using the all-MiniLM-L12-v2 model,^56^ a lightweight transformer-based embedding model designed for semantic similarity tasks.

The model was selected following a two-stage validation approach to assess different aspects of embedding performance of various language model variants. The first stage evaluated broad topic clustering accuracy using a corpus of 11,676 documents spanning seven distinct research domains. For each domain, one document was randomly selected as a query, embedded using each candidate model, and precision@1000 was calculated by measuring the percentage of retrieved documents that belonged to the same research domain as the query according to predefined domain labels. This stage assessed the models’ capacity to distinguish between fundamentally different research areas at scale. Results showed MiniLM-L12 achieved 92.75% precision@1000, slightly below Specter^57^ (93.8%) and MPNet^58^ (93.1%) but substantially outperforming other alternatives.

Stage 2 evaluated fine-grained similarity detection using a smaller gold-standard dataset of 116 documents manually labeled across seven overlapping research subtopics within the same broad field. For each subtopic, one document was randomly selected as a query, and cosine similarity was calculated between the query embedding and all 116 document embeddings in the dataset. Precision@10 was measured by counting how many of the top 10 retrieved documents belonged to the same subtopic as the query document. While this represents a relatively small evaluation set, it provided a comparative assessment of the models’ ability to distinguish between closely related but distinct research approaches.

Stage 2 results revealed important differences: MiniLM-L12 achieved 82.8% precision@10, comparable to MPNet (84.2%) and significantly better than Specter (67.1%). Computational efficiency testing with 5,000 abstracts showed substantial speed differences, with MiniLM-L12 processing documents in 2 min 21 s compared to 17 min 4 s for Specter and 16 min 7 s for MPNet. Given the requirement to process 20 million abstracts, MiniLM-L12 was selected as it provided the optimal balance of semantic accuracy and computational efficiency for large-scale document matching.

### Matching algorithm

For each abstract, the text was tokenized through the MiniLM encoder, and the resulting embedding vector was obtained. These vector representations were then indexed in Elasticsearch, which efficiently implements an approximate nearest neighbor search using an inverted file index with product quantization (IVFPQ). Each SDG 13 article was compared against 20 million Scopus articles using cosine similarity as the distance metric. Only matches with a minimum cosine similarity threshold of 80% were retained, as this threshold was empirically determined to balance precision with recall.

**Figure 9:**
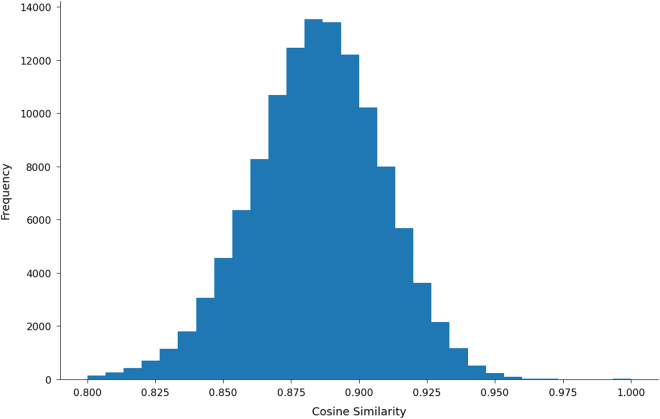
Cosine similarity distribution of filtered matched pairs

To ensure the relevance and comparability of the matched articles, the first two steps of the filtering approach by Bikard and Marx^59^ were applied. First, pairs published more than one year apart were removed, refining the dataset to 65,545 queried articles matched with 182,959 documents. Second, matches with authors in common were excluded, further refining the dataset to 57,241 queried articles matched with 77,808 similar articles. Cosine similarities of all matches clustered around 0.88, with a standard deviation of 0.02, as displayed in Figure 9 below.

We then identified which of these papers were cited in policy documents indexed in Overton and found 3,385 pairs where both papers were cited in policy, 67,279 pairs where neither paper was cited in policy, and 13,992 “near misses,” where one paper was cited in policy and the other was not.

**Figure 10:**
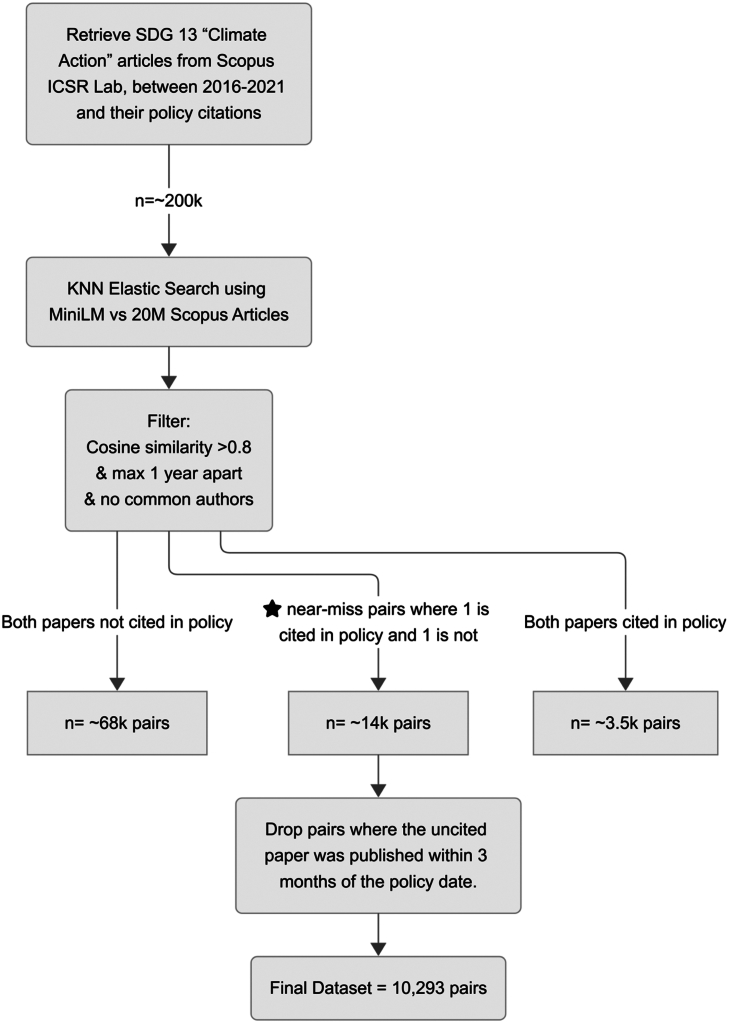
Workflow for near-miss pair selection

The near-misses dataset was further refined by excluding pairs where the non-cited article was published after the citing policy document publication date, resulting in a final dataset of 10,293 pairs, comprising 8,454 distinct cited papers matched with 9,843 distinct, not cited “near misses.” A flowchart describing the methodology is shown in in Figure 10 below.

Manual validation of 50 randomly selected pairs was conducted by a member of the research team with domain expertise in climate policy. The reviewer assessed whether (1) both papers addressed the same climate policy issue and (2) both papers provided equivalent types of evidence that would be comparably useful for policymakers working on that issue. Pairs were classified as false positives if papers addressed fundamentally different topics or if one paper was clearly more policy relevant than its match. No false positives were identified in the sample, confirming that matched pairs represent cognate research addressing equivalent policy questions. A sample of cited and near-miss paper titles are displayed in Table 1, while their abstracts are provided in Table S1.

**Table 1:** Sample of cited and near-miss paper titles

Cited title	Near-miss title
Black Carbon Emissions and Associated Health Impacts of Gas Flaring in the United States	Quantification of the global and regional impacts of gas flaring on human health via spatial differentiation
Characterizing particulate matter emissions from GDI and PFI vehicles under transient and cold start conditions	Field Measurements of Gasoline Direct Injection Emission Factors: Spatial and Seasonal Variability
Modeling household energy consumption and adoption of energy efficient technology	The electricity footprint of household activities—implications for demand models
Help the climate, change your diet: A cross-sectional study on how to involve consumers in a transition to a low-carbon society	A Survey of Registered Dietitians’ Concern and Actions Regarding Climate Change in the United States
Advances in the thermo-chemical production of hydrogen from biomass and residual wastes: Summary of recent techno-economic analyses	Production of biohydrogen from gasification of waste fuels: Pilot plant results and deployment prospects

While most cited papers had only one near-miss match, approximately 17% of the cited papers had more than one match. Fifteen outliers had more than five matches. The distribution of matches is shown in Table 2. For this analysis, even though the cited papers with multiple matches are duplicated, each cited/near-miss match is treated as an independent pair.

**Table 2:** Frequency of papers by number of near-miss matches

Number of near-miss matches per cited paper	Frequency
1	7,006
2	1,142
3	244
4	47
5	10
6	3
7	1
8	1

Furthermore, while almost 82% of the matches are independent pairs, the remaining 18% form interconnected clusters of up to 12 papers, as shown in Table 3. For the purposes of this analysis, we also treat each pair as independent, regardless of whether it forms part of a larger interconnected cluster.

**Table 3:** Frequency of cluster size

Cluster size	No. clusters
2	6,323
3	1,285
5	73
6	19
7	12
8	2
9	1
10	2
12	1

Since the near-misses dataset includes pairs of highly similar research papers, where one is cited in policy and its counterpart is not, we proceeded to evaluate the influence of different indicators on the likelihood of policy citation, controlling for the confounding effect of their content’s inherent policy relevance. For each paper, we extracted a set of indicators related to academic citations, co-authorship with government, past policy citations, altmetrics, and geography. For each of these indicators, we standardized the continuous variables into *Z* scores and perform a series of conditional logistic regressions using the “survival” package in R.^60^ The models allow us to evaluate the effect of each indicator on the likelihood of a paper being cited in policy documents, while controlling for confounding factors inherent to the paired papers.

Across all models, we examine the coefficients to determine the strength and direction of each indicator’s impact, standard errors to assess the precision of these estimates, and *p* values to confirm statistical significance. We also review odds ratios to understand the magnitude of the effects and compare model fit statistics to evaluate the overall effectiveness of the models.

Our methodology offers a more scalable approach to controlling for latent characteristics of research content compared to previous work by Marx and Hsu.^22^ Rather than manually identifying research twins through shared citation patterns, our computational approach using semantic similarity enables broader analysis without requiring access to full-text data or laborious manual examination or specialized language models for each field.

## Resource availability

### Lead contact

Requests for further information and resources should be directed to and will be fulfilled by the lead contact, Basil Mahfouz (basil.mahfouz.21@ucl.ac.uk).

### Materials availability

This study does not contain any new materials.

### Data and code availability

Scopus data-sharing policies prohibit sharing the underlying 20 million article database used for the KNN semantic matching. The embedding generation and KNN matching infrastructure requires Elsevier’s proprietary systems and underlying data. We provide the final matched dataset of 10,293 paper pairs with DOIs, titles, bibliometric indicators, and all analytical code (PySpark and R notebooks) on GitHub (https://github.com/patterns-nearmiss/climate-policy-casestudy), which have been archived at Zenodo.^61^ Original matching pipeline and raw database access may be available upon request with Elsevier’s permission. Contact Dr. Andrew Plume (mailto:a.plume@elsevier.com).

## Acknowledgments

We acknowledge Elsevier for funding, technical support, data access, and computational resources. We thank Dr. Andrew Plume for strategic guidance and facilitating access to necessary resources, Kristy James for technical expertise and methodological input, and Alick Bird, Colin Ke Han Zhang, and Tian Mahony for infrastructure implementation.

## Author contributions

Conceptualization, B.M., L.C., and G.M.; methodology, B.M. and L.C.; software, B.M.; validation, B.M.; formal analysis, B.M., L.C., and G.M.; investigation, B.M.; data curation, B.M.; writing – original draft, B.M.; writing – review & editing, L.C. and G.M.; visualization, B.M.; supervision, L.C. and G.M.; project administration, B.M. and G.M.

## Declaration of interests

The authors declare no competing interests.

## Declaration of generative AI and AI-assisted technologies in the writing process

During the preparation of this work, the authors used Anthropic AI Claude 3.7 Sonnet to edit the language style in some areas and to generate the correct reference syntax. After using this tool, the authors reviewed and edited the content as needed and take full responsibility for the content of the publication.

## Supplemental information

Table S1. Sample of near-miss abstracts

Document S1. Article plus supplemental information
