# Supplementary material for: Uncovering drivers of climate research in policy with pretrained language models
Source: Patterns (N Y). 2025 Aug 7;6(11):101342. doi: 10.1016/j.patter.2025.101342 (PMC12664962; doi:10.1016/j.patter.2025.101342)
Supplement: Table S1. Sample of near-miss abstracts [file mmc1.pdf]

Patterns, Volume *6*

Supplemental information

Uncovering drivers of climate research in policy with pretrained language models

Basil Mahfouz, Licia Capra, and Geoff Mulgan

Table S1. Sample of near miss Abstracts

	Policy-cited Abstract	Near Miss Abstract
1	Gas flaring from oil and gas fields is a significant source of black carbon (BC) emissions, a component of particulate matter that damages health and warms the climate. Observations from the Visible Infrared Imaging Radiometer Suite (VIIRS) satellite instrument indicate that approximately 17.2 billion cubic meters (bcm) of gas was flared from upstream oil and gas operations in the United States in 2019. Based on an emissions factor equation that accounts for the higher heating value of the gas, that corresponded to nearly 16,000 tons of BC emitted, though estimates vary widely across published emissions factors. In this study, we used three reduced-form air quality and health effect models to estimate the health impacts from the flaring-emitted BC particulate matter in the United States. The three models—EASIUR, AP3, and InMAP—predict 26, 48, and 53 premature deaths, respectively, in 2019. The mortality range expands from 5 to 360 deaths annually if alternative emission factors are used. This study shows that reduced-form models can be useful to estimate the impacts of numerous dispersed emissions sources such as flares, and that further research is needed to better quantify BC emissions factors from flares.	Globally, gas flaring caused 350 million tonnes of CO_2 emissions in 2018. In addition to climate change, this burning practice also has other negative consequences for humans (e.g., respiratory problems) and the environment. The aim of this paper is to quantify the impact of flaring on human health (at the global and country level) via the calculation of the number of disability-adjusted life years (DALYs) caused by the combustion of associated gas. For this quantification, gas flaring emissions were linked with midpoint indicators (e.g., climate change) in life cycle assessment (LCA) for all selected countries. Regionalised characterisation factors (CFs) were applied in the impact assessment to allow for spatial differentiation. The global impact on human health caused by flaring was obtained by taking the sum for all countries. The results show that these flaring emissions globally cause 4.83 10^5 DALYs or 6.19 10^{-5} DALYs/person on an annual basis. This amounts to 0.12% of the total DALYs related to air pollution (from $PM_{2.5}$) caused by all polluting sectors and 6.51% of the total DALYs related to climate change. To quantify these impacts, this study uses a country perspective rather than considering local characteristics. Thus, if more precise information at a more local level (e.g., city level) is sought, additional factors (e.g., meteorological conditions) should be taken into account. Finally, future research should also focus on the benefits of gas flaring reduction techniques to enable the selection of the most promising technologies for the elimination of gas flaring and its effects.
2	Ultrafine particle emissions from Gasoline Direct Injection (GDI) and Port Fuel Injection (PFI) vehicles under the New European Driving Cycle (NEDC) were measured by using the Differential Mobility Spectrometer 500 (DMS 500) for size distribution information and Transmission Electronic Microscopy (TEM) for morphological analysis. The first phase of the ECE 15 cycle (representing the urban driving condition) within the NEDC was examined to evaluate the cold start effect on particulate matter (PM) emissions. Even though the ECE 15 cycle1 only occupies 1/6th of the time of the NEDC, the cold start emissions accounted for more than 50% of the	Four field campaigns were conducted between February 2014 and January 2015 to measure emissions from light-duty gasoline direct injection (GDI) vehicles (2013 Ford Focus) in an urban near-road environment in Toronto, Canada. Measurements of CO_2, CO, NO_x, black carbon (BC), benzene, toluene, ethylbenzene-xylenes (BTEX), and size-resolved particle number (PN) were recorded 15 m from the roadway and converted to fuel-based emission factors (EFs). Other than for NO_x and CO, the GDI engine had elevated emissions compared to the Toronto fleet, with BC EFs in the 73rd percentile, BTEX EFs in the 80–90th percentile, and PN EFs in the 75th

	total particle number for GDI vehicles and approximately 70% for PFI vehicles. Further analysis in terms of the Count Medium Diameter (CMD) of the PM emissions is also discussed. A linear regression method was adopted to analyze the correlation between PM emissions and vehicle parameters. For GDI vehicles, acceleration demonstrated a strong correlation with PM emissions, particularly under warm start conditions. The effects of the after treatment device Three Way Catalyst (TWC) on PM emissions was also investigated.	percentile during wintertime measurements. Additionally, for three campaigns, a second platform for measuring PN and CO_2 was placed 1.5–3 m from the roadway to quantify changes in PN with distance from point of emission. GDI vehicle PN EFs were found to increase by up to 240% with increasing distance from the roadway, predominantly due to an increasing fraction of sub-40 nm particles. PN and BC EFs from the same engine technology were also measured in the laboratory. BC EFs agreed within 20% between the laboratory and real-world measurements; however, laboratory PN EFs were an order of magnitude lower due to exhaust conditioning.
3	This study develops a model to study household energy use behavior that can impose common preferences for feasible demand estimation with multiple discrete technology choices and multiple continuous energy consumption uses. The model imposes fixed proportions production and additivity of uses for plausible estimation feasibility while adopting a second-order translog flexible functional form to focus on flexibility in identification of consumer preferences that determine interactions among energy uses and between short-run and long-run choices. Using a unique household-level dataset from California, the model is applied to estimate short-run household demand for electricity and natural gas and the long-run technology choices with respect to clothes washing, water heating, space heating, and clothes drying. The estimation results support commonality of underlying preferences except in one case that is explained by an unavailable variable.	It is an intuitive assumption that some activities require more energy than others. Bottom-up energy demand models therefore use time-use data to inform the timing of energy use. In this paper we present some empirical evidence to test the strength of this assumption.<br>Using data that simultaneously captures household activities and their coinciding electricity consumption, it is possible to relate one to the other. We validate the temporal accuracy of the approach with the example of reporting hot drinks and the distinct signature of kettle usage. Despite good data accuracy, the predictive power of reported activities for electricity use is modest. At time when activities that would subjectively be associated with high energy consumption are reported, electricity use is only about 7% higher than at times with activities of low energy association. For single occupant households the link is stronger with more than 30% difference between the two activity categories.<br>We conclude that demand models may need to take account of diversity and complexity in multi-occupant households and that more sophisticated regression techniques may be required to improve demand predictions based on time-use data.
4	This paper explores how the transition to a low-carbon society to mitigate climate change can be better supported by a diet change. As climate mitigation is not the focal goal of consumers who are buying or consuming food, the study highlighted the role of motivational and cognitive background factors, including possible spillover effects. Consumer samples in the Netherlands (n = 527) and the United States (n = 556) were	Dietary choices are a tool to reduce greenhouse gas emissions. While registered dietitians are on the front lines of food and nutrition recommendations, it is unclear how many are concerned with climate change and take action in practice in the United States. We explored concern about climate change among registered dietitians, and identified factors that may influence practice-related behaviors. Our study

	asked to evaluate food-related and energy-related mitigation options in a design that included three food-related options with very different mitigation potentials (i.e. eating less meat, buying local and seasonal food, and buying organic food). They rated each option's effectiveness and their willingness to adopt it. The outstanding effectiveness of the less meat option (as established by climate experts) was recognized by merely 12% of the Dutch and 6% of the American sample. Many more participants gave fairly positive effectiveness ratings and this was correlated with belief in human causation of climate change, personal importance of climate change, and being a moderate meat eater. Willingness to adopt the less meat option increased with its perceived effectiveness and, controlling for that, it was significantly related to various motivationally relevant factors. The local food option appealed to consumer segments with overlapping but partly different motivational orientations. It was concluded that a transition to a low carbon society can significantly benefit from a special focus on the food-related options to involve more consumers and to improve mitigation.	population included a random sample of all registered dietitians credentialed in the United States. Primary data were gathered using a cross-sectional survey. Of the 570 survey responses, 75% strongly agreed or agreed that climate change is an important issue while 34% strongly agreed or agreed that dietitians should play a major role in climate change mitigation strategies. Thirty-eight percent engaged in activities that promoted diet as a climate change mitigation strategy. Vegetarian ($p = 0.002$) and vegan dietitians ($p = 0.007$) were significantly more likely than non-vegetarian and non-vegan dietitians to engage in activities that promoted diet as a climate change mitigation strategy. Overall, concern for climate change among dietitians varied significantly by the region of the country in which the dietitian resided, and awareness that animal products are implicated in climate change. Registered dietitians in the United States are concerned with climate change. However, there is a discrepancy between concern and practice-based actions. These results suggest the need for educational and experiential opportunities connecting climate change mitigation to dietetics practice.
5	This article outlines the prospects and challenges of hydrogen production from biomass and residual wastes, such as municipal solid waste. Recent advances in gasification and pyrolysis followed by reforming are discussed. The review finds that the thermal efficiency of hydrogen from gasification is ~50%. The levelized cost of hydrogen (LCOH) from biomass varies from ~2.3–5.2 USD/kg at feedstock processing scales of 10 MWth to ~2.8–3.4 USD/kg at scales above 250 MWth. Preliminary estimates are that the LCOH from residual wastes could be in the range of ~1.4–4.8 USD/kg, depending upon the waste gate fee and project scale. The main barriers to development of waste to hydrogen projects include: waste pre-treatment, technology maturity, syngas conditioning, the market for clean hydrogen, policies to incentivize pioneer projects and technology competitiveness. The main opportunity is to produce low cost clean hydrogen, which is competitive with alternative production routes.	Hydrogen is seen as a key element of the future energy mix because it does not generate greenhouse gas emissions at the point of use. Understanding the technologies that can generate low carbon hydrogen is essential in planning the development of future gas networks and energy generation via fuel cells. One promising approach is hydrogen production by gasification of waste, referred to as biohydrogen. This paper summarises work undertaken to design a commercial Waste-to-Hydrogen (WtH_2) plant, which includes an assessment of future markets for hydrogen, the identification of an appropriate scale for the plants, and development of specifications for process design and output streams. An experimental programme was undertaken to demonstrate $bioH_2$ production from refuse derived fuel (RDF) at pilot scale and provided experimental data to underpin commercial designs. On this basis, a reference design for small commercial plants was developed for $bioH_2$ production for heating and transport utilisation. A preliminary carbon assessment shows that carbon savings for biohydrogen in a commercial scale are more than four times greater than alternative technologies.
